# Pyoverdine, a siderophore from *Pseudomonas aeruginosa*, translocates into *C. elegans*, removes iron, and activates a distinct host response

**DOI:** 10.1080/21505594.2018.1449508

**Published:** 2018-04-24

**Authors:** Donghoon Kang, Daniel R. Kirienko, Phillip Webster, Alfred L. Fisher, Natalia V. Kirienko

**Affiliations:** a Department of BioSciences, Rice University, Houston TX , USA; b Center for Healthy Aging, University of Texas Health Sciences Center, San Antonio TX , USA; c GRECC, South Texas VA Healthcare System, San Antonio TX , USA

**Keywords:** pyoverdine, siderophore, mitochondrial damage, host response, *Pseudomonas aeruginosa*, *Caenorhabditis elegans*, pathogenesis

## Abstract

*Pseudomonas aeruginosa*, a re-emerging, opportunistic human pathogen, encodes a variety of virulence determinants. Pyoverdine, a siderophore produced by this bacterium, is essential for pathogenesis in mammalian infections. This observation is generally attributed to its roles in acquiring iron and/or regulating other virulence factors. Here we report that pyoverdine translocates into the host, where it binds and extracts iron. Pyoverdine-mediated iron extraction damages host mitochondria, disrupting their function and triggering mitochondrial turnover via autophagy. The host detects this damage via a conserved mitochondrial surveillance pathway mediated by the ESRE network. Our findings illuminate the pathogenic mechanisms of pyoverdine and highlight the importance of this bacterial product in host-pathogen interactions.

## Introduction

*Pseudomonas aeruginosa* is a major nosocomial bacterial pathogen that causes a variety of infections, particularly in patients with disorders leading to compromised immune systems and those with cystic fibrosis or significant burn wounds[1,2]. To drive infection in these disparate conditions, *P. aeruginosa* utilizes a diverse armamentarium of virulence factors, including those involved in colonization and infection (flagella, pili, *etc*.), nutrient acquisition (phospholipases, exoproteases, siderophores), and various modulators of host response.

Despite intense study, novel roles for many key virulence determinants are still being identified. For example, it was recently discovered that pyoverdine, a siderophore produced by *P. aeruginosa*, is required for pathogenesis in *Caenorhabditis elegans* [3,4] and many mammalian infection models [5,6,7,8]. Small molecules that inhibit its biosynthesis have been shown to effectively limit *P. aeruginosa* pathogenesis, providing a promising approach to tackle antimicrobial resistance in this microorganism [7,9,10,11].

Pyoverdine has several known functions that contribute to virulence. It acts as an important (and sometimes irreplaceable) route for acquisition of ferric iron, a nutrient essential for growth. Pyoverdine binds ferric iron in a 1:1 stoichiometric ratio with an exceptionally high affinity, which is sufficient to extract the metal from mammalian iron-sequestering proteins such as transferrin [5]. By providing iron to the pathogen, pyoverdine can also contribute to the formation of biofilms [5]. Finally, bacterial recognition of the iron:pyoverdine complex (known as ferripyoverdine) by its receptor, FpvA, derepresses PvdS [13]. PvdS is an alternative sigma factor whose targets include the protease PrpL, the translational inhibitor ToxA, and the biosynthetic machinery for pyoverdine itself, leading to a positive feedback loop [14,15,16,17]. The resulting amplification of pyoverdine production is terminated only when intracellular iron demand is satiated [18]. The accepted paradigm for the importance of pyoverdine in host pathology has generally been attributed to some combination of these activities. Our recent discovery that pyoverdine can kill *C. elegans*, even in the absence of the bacterial pathogen [3], indicates that this paradigm must be expanded. However, the mechanism of pyoverdine-mediated pathology has remained elusive.

In this report, we demonstrate that pyoverdine transiently enters the host and removes significant amounts of ferric iron. As a consequence of this activity, mitochondria suffer damage, compromising electron transfer and ATP production, ultimately activating mitochondrial turnover. *C. elegans* detects this damage, likely through mitochondrial surveillance, and initiates a specific host response distinct from defense against *P. aeruginosa* infection.

## Results and discussion

### Pyoverdine translocates into C. elegans

**Figure 1.**
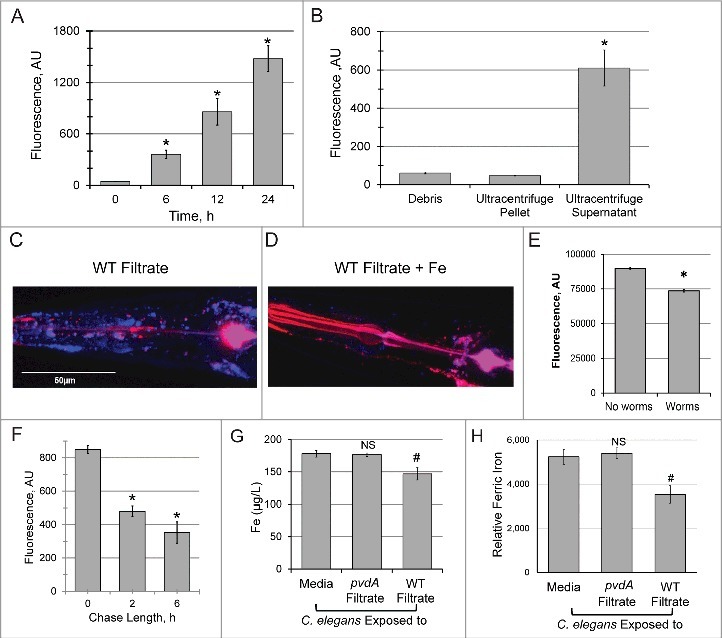
Pyoverdine translocates into *C. elegans*, extracts host iron, and exits the host. (A) Pyoverdine fluorescence was measured in *C. elegans* lysates. Worms were exposed to pyoverdine-rich filtrate for the indicated amount of time, washed, and then lysed. Complexed iron was stripped from pyoverdine using 1M 8-hydroxyquinoline before measurement. (B) Pyoverdine fluorescence was measured in *C. elegans* cellular debris, cellular membranes, or soluble protein fractions. (C, D) Maxmium projection of confocal laser scanning microscopy images of pyoverdine fluorescence in *glp-4(bn2)* mutant worm tissue after exposure to pyoverdine-rich filtrate (C) or the same filtrate pre-saturated with iron (D). Blue indicates fluorescence of iron-free pyoverdine, red shows CellMask plasma membrane stain. Images were collected using identical settings. (E) Fluorescence of pyoverdine-rich filtrate after 24h incubation in the presence or absence of *C. elegans*. (F) Pyoverdine fluorescence in the lysates of worms exposed to pyoverdine for 16 h (pulse), and then incubated in pyoverdine-free medium for 0, 2, or 6 h (chase). Total levels of pyoverdine were measured after complexed iron was extracted with 1M 8-hydroxyquinoline. (G) ICP-MS analysis of total iron concentration in worms exposed to either pyoverdine-rich filtrate, filtrate from a pyoverdine biosynthesis mutant *pvdA*, or media control. (H) Relative amount of ferric iron in the lysates of worms exposed to filtrate from pyoverdine-free media (*E. coli* OP50), pyoverdine-rich filtrate from wild-type *P. aeruginosa*, or filtrate from a *P. aeruginosa pvdA*. Asterisks or hashes indicate significant difference between conditions (*p*-value < 0.01 or < 0.05, correspondingly) based on Student's *t*-test. Error bars represent SEM of three independent biological replicates.

We previously reported detection of pyoverdine within *C. elegans* after the host was exposed to the substance for prolonged periods [3]. To gain additional insight into this phenomenon, we took advantage of the characteristic fluorescence of pyoverdine to assay its accumulation. In brief, worms were exposed to pyoverdine-rich, cell-free spent bacterial growth media (referred to hereafter as filtrate, see Methods for details for this and other procedures) for several hours to allow pyoverdine to enter the host. Afterward, worms were extensively washed to remove pyoverdine outside the host and lysed to release intracellular contents. Fluorescence was then measured via spectroscopy. Because pyoverdine fluorescence is largely quenched when it binds iron, it is necessary to remove the metal to accurately determine the siderophore concentration inside of the worm. Treatment with excess 8-hydroxyquinoline (8HQ) strips iron from pyoverdine, restoring fluorescence [19] and allowing us to measure total pyoverdine within the host to be measured. Using this technique, we observed a time-dependent increase in pyoverdine fluorescence within the host up to 24h (Fig 1A). To determine whether the pyoverdine observed was associated with cell membranes (*i.e.*, it is attached to the exterior of the cells or has bound a receptor), *C. elegans* were disrupted via sonication and subjected to ultracentrifugation to separate cell debris and microsomes from cytoplasmic content. Pyoverdine was observed only in the soluble protein fraction, which corresponds to cytoplasmic content (Fig 1B), suggesting that the majority of the pyoverdine within the host is not associated with cellular membranes.

To confirm that pyoverdine was located within the worms, we used confocal laser-scanning microscopy to visualize pyoverdine. *glo-1*(*zu391*) worms were used, as this mutation decreases the number of autofluorescent gut granules (which have fluorescence profile similar to pyoverdine) [20]. Even in this mutant background, significant intestinal autofluorescence remained, so we restricted our analysis to the pharyngeal and tail regions, where autofluorescent granules are absent. Pyoverdine fluorescence was clearly observed in the extralumenal tissues surrounding the pharnyx and in the tail (Fig 1C, Fig S1, Movie S1). This fluorescence was quenched by the addition of exogenous iron(III) and enhanced by supplementation with gallium(III) prior to worm exposure [21]. These changes in fluorescence are characteristic of pyoverdine (Fig 1C, Fig S1, Movie S1). *glo-1* worms were also exposed to identically-produced filtrate from a pyoverdine biosynthesis mutant (*P. aeruginosa* PA14*pvdA*) or to uninoculated media (Fig S1, Movie S1). None of these conditions showed fluorescence inside *C. elegans* pharynx or tail, although weak fluorescence was observed in the intestinal lumen in some cases.

Although we saw a clear difference in pyoverdine fluorescence between worms exposed to pyoverdine-rich media and all other conditions, the fluorescence we observed likely underrepresents the amount of pyoverdine within the host. Upon entering *C. elegans*, pyoverdine binds host iron (see below), quenching its fluorescence. In contrast, complexing pyoverdine with gallium(III) improves pyoverdine's quantum yield [21] and reduces pyoverdine's sensitivity to ironquenching. We took advantage of these phenomena to enhance the fluorescence of intraorganismal pyoverdine, which dramatically improved its visualization (Fig S1).

Another way to test whether pyoverdine has access to intra-organismal iron is to incubate pyoverdine-rich filtrate with worms and see if this decreases fluorescence. For this purpose, identical aliquots of pyoverdine-rich filtrate were prepared and *C. elegans* was added to one sample. After 24 h, worms were removed by centrifugation, and fluorescence in the filtrate was measured. Fluorescence of filtrate exposed to worms was significantly reduced compared to the untreated control (Fig 1E). It is important to note that this difference likely reflects two separate mechanisms of fluorescence loss: first, since worms were removed from the filtrate, any pyoverdine that has been internalized by the host will be lost. Second, binding of host iron also quenches fluorescence.

Since pyoverdine's function is to acquire iron for the pathogen, we hypothesized that the ferripyoverdine, now carrying iron liberated from the host, would make its way back out of the host and into the media. Given the high concentration of pyoverdine in the media and the comparatively low amount that enters the host, it is unlikely we could make sufficiently accurate measurements to conclusively demonstrate pyoverdine egress. Therefore, we assayed pyoverdine fluorescence (after 8HQ treatment) within the host instead, using a pulse-chase methodology. Worms were loaded with siderophore by exposing them to pyoverdine-rich filtrate for 16 h and either washed and frozen immediately or transferred to pyoverdine-free medium for 2 or 6 h to permit the siderophore to exit the host. Within the first 2 h of the chase, over 40% of pyoverdine was lost; this loss continued, more gradually, until at least 6 h (Fig 1F).

### Pyoverdine exposure reduces iron content of C. elegans

We used inductively coupled plasma mass spectrometry (ICP-MS) to quantitatively measure the impact of pyoverdine translocation on the total iron pool in *C. elegans*. Exposure to pyoverdine-rich filtrate reduced the host iron content by 17.5% (*p*<0.05), while identically-produced filtrate from *P. aeruginosa* PA14*pvdA*, a strain deficient in pyoverdine biosynthesis, had no effect on the host iron pool (Fig 1G).

Because pyoverdine fluorescence is quenched by iron binding, pyoverdine can be used as a sensitive, relatively specific, quantitative, fluorometric reporter of iron concentration. This method selectively measures oxidized (ferric) iron, as pyoverdine has a very weak affinity for iron(II).

We took advantage of this trait to measure pyoverdine-mediated removal of ferric iron from *C. elegans*. In brief, we exposed equal numbers of worms to filtrate from *P. aeruginosa* (pyoverdinerich), to filtrate from *P. aeruginosa* PA14*pvdA* (pyoverdine deficient), or media control. After exposure, worms were extensively washed with pyoverdine-free media and then lysed to release all available iron. Each sample was then mixed with a known quantity of pyoverdine, which would be partially quenched by the iron in the lysate. As the stoichiometric relationship of pyoverdine and iron is 1:1, the difference between initial and final pyoverdine fluorescence reflects the relative amount of ferric iron present in each sample (Fig 1H; see Fig S2 for raw data).

Worms treated with filtrate from wild-type bacteria retained 32.6% less ferric iron than worms treated with media control (*p*<0.05). In contrast, worms treated with filtrate from *P. aeruginosa* PA14*pvdA* were indistinguishable from those treated with media. It should be noted that the apparent discrepancy between measures of ferric (∼32.6%, fluorometric analysis) and total iron (∼17.5%, ICP-MS) is unlikely to reflect a true difference; the former technique only measures iron(III). Measurements of ferric iron are more likely to be the relevant metric, as Fe^3+^ is the natural ligand for pyoverdine (making it more likely to be liberated from the host).

It is important to note that the PA14*pvdA* mutant did not exhibit growth defects in the media used for this assay (Fig S3A-C), reducing the likelihood that the observed differences between wild-type PA14 and PA14*pvdA* were due to an inability of the mutant to grow in this medium.

To validate the physiological relevance of our findings, we also tested the ability of pyoverdine produced by living *P. aeruginosa*, under assay conditions, to translocate into the host and extract iron. In each case, phenomena observed with live bacteria recapitulated our observations with filtrate (Fig S4A-C).

We tested whether two synthetic iron chelators (1,10-phenanthroline and ciclopirox olamine) could recapitulate our observations with pyoverdine. Neither compound reduced host iron content, as assessed by ICP-MS (Fig S5A-B). This was surprising for two reasons. First, these compounds are known to damage mitochondria, likely by removing iron [3,22,23], suggesting that they must also get into the host under some conditions, including those tested here. Second, the addition of phenanthroline to *C. elegans* resulted in the formation of a reddish material (Fig S5C), which is characteristic of a phenanthroline:iron complex [24]. Since sample preparation for ICP-MS involves complete acid hydrolysis of the biological material, we hypothesize that these chelators formed complexes with the hosts' iron that were unable to exit the host. This would lead to the reported mitochondrial damage and accumulation of the reddish color while also explaining the ICP-MS measurements observed.

### Exposure to pyoverdine damages host mitochondria

**Figure 2.**
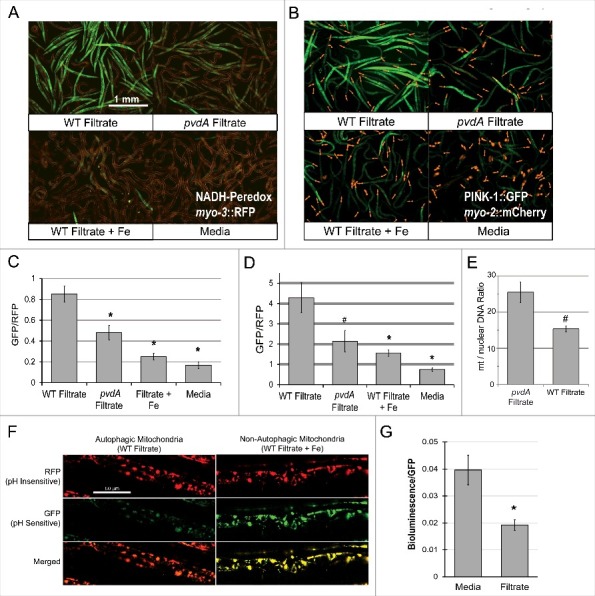
Exposure to pyoverdine damages host mitochondria. (A, B) Visualization of a Peredox fluorescence reporter bound to NADH, normalized to a constitutively-expressed, RFP-tagged protein (A) or GFP fluorescence from a PINK-1::GFP reporter, normalized to a constitutively-expressed, RFP-tagged protein (B). Worms were exposed to pyoverdine-rich or *pvdA* filtrate, pyoverdine-rich filtrate saturated with iron, or media. (C, D) Quantification of fluorescence for the conditions above. At least 150 worms were analyzed for each biological replicate (approximately 50 worms per image). (E) Relative quantification of mitochondrial:nuclear genome copy number in worms exposed to pyoverdine-rich filtrate or filtrate from PA14*pvdA* (control). (F) Fluorescence of the mtRosella reporter in worms treated with wild-type filtrate or wild-type filtrate pre-saturated iron. The GFP signal is sensitive to low pH, and loses fluorescence within autophagolysosomes. (G) Bioluminescence of a GFP-tagged luciferase in *C. elegans* exposed to pyoverdine-rich filtrate or media control. Light and GFP fluorescence were measured 1 hour after the addition of luciferin. Luminescence was normalized to GFP fluorescence to control for differences in protein expression. Data presented in A, B, F, and G are one representative result from four biological replicates. Data presented in E are the average from three biological replicates. Error bars in C, D, E, G represent SEM. Asterisks indicate *p*-value < 0.01, hashes are *p* < 0.05, based on Student's *t*-test.

Host mitochondria represent a rich intracellular pool of iron, albeit one comprised of integral iron-sulfur cluster proteins responsible for a variety of metabolic activities. One key function of mitochondrial iron-sulfur proteins is the generation of ATP via oxidative phosphorylation. This process involves the oxidation of NADH to NAD^+^, making the ratio of these metabolites a sensitive indicator of mitochondrial function. Using a Peredox-based reporter that fluoresces upon binding NADH (but not NAD^+^) [25], we measured the cytosolic NADH:NAD^+^ ratio to assess mitochondrial function after exposure to pyoverdine-rich filtrate, and saw substantial accumulation of the reduced form (Fig 2A,C). The NADH:NAD^+^ ratio was significantly lower in worms treated with filtrate from PA14*pvdA*, iron-bound pyoverdine, or media.

Mitochondria generally lack significant repair capacity; instead, damaged or excess mitochondria are recycled via autophagic turnover in a process mediated by the kinase PINK-1/PINK1 [26]. This protein is constitutively expressed and is rapidly imported into the matrix of healthy mitochondria, where it is destroyed by resident proteases [27]. Mitochondrial damage compromises protein import, increasing PINK-1/PINK1 stability. This change can be readily observed in *C. elegans* by using a GFP-tagged PINK-1/PINK1 construct. Therefore, we exposed a strain carrying PINK-1/PINK-1::GFP to pyoverdine-rich filtrate (Fig 2B,D). We observed strong stabilization of PINK-1/PINK1, indicating that pyoverdine treatment has compromised the mitochondrial membrane potential, and/or protein import.

Under normal physiological conditions, mitochondrial synthesis and turnover are carefully balanced to maintain cellular homeostasis. However, we hypothesized pyoverdine exposure is likely to disturb this balance, tilting it toward mitochondrial turnover. To test this, we measured the ratio of mitochondrial-to-nuclear genome counts in worms treated with either pyoverdine-rich filtrate or filtrate from a pyoverdine-deficient control (Fig 2E). As expected, pyoverdine treatment reduced this ratio, indicating that damaged mitochondria are being recycled.

To determine whether mitochondria are being turned over via autophagy, we used a mitochondrially-targeted Rosella construct [28]. In brief, this reporter takes advantage of the relative instability of GFP at acidic pH, as compared to the more stable DsRed protein. A *C. elegans* strain expressing this reporter was exposed to either wild-type filtrate, filtrate from PA14*pvdA*, wild-type filtrate pre-saturated with ferric iron, or to media alone (Fig 2F, Fig S6). GFP and DsRed fluorescence were visualized by confocal laser-scanning microscopy. Only wild-type filtrate triggered mitophagy as demonstrated by the diminished GFP fluorescence, indicating fusion of the mitochondria-containing autophagosomes with lysosomes. This phenomenon was not seen if pyoverdine was pre-saturated with ferric iron.

As a final measurement of mitochondrial function, we used a luciferase reporter to assay ATP content in worms exposed to pyoverdine. Using a strain of *C. elegans* expressing a GFP-tagged luciferase (which allows luminescence to be normalized to GFP fluorescence), we measured cellular ATP content in worms exposed to either media alone or pyoverdine-rich filtrate. We observed that pyoverdine exposure significantly decreased cellular ATP content (Fig 2G), suggesting that pyoverdine-mediated mitochondrial damage compromises ATP production.

**Figure 3.**
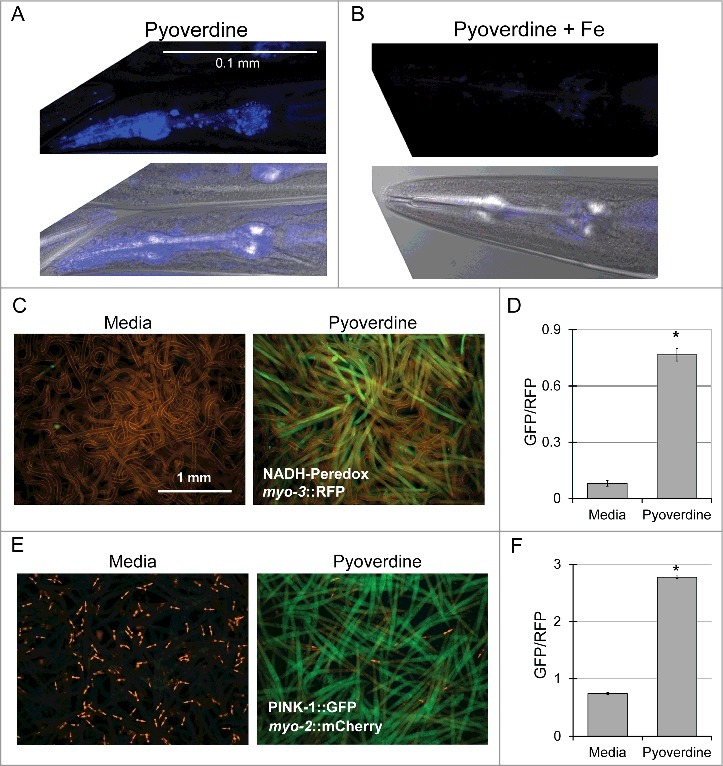
Purified pyoverdine translocates into the host and activates mitophagy. (A, B) Confocal laser scanning microscopy images of pyoverdine fluorescence in *glp-4(bn2)* mutant worm tissue after exposure to purified pyoverdine without (A) or with (B) iron supplementation. Images were collected using identical settings. (C, D) Visualization (C) and quantification (D) of a Peredox fluorescence reporter normalized to a constitutively-expressed, RFP-tagged protein. Worms were exposed to purified pyoverdine or media. (E, F) Visualization (E) and quantification (F) of a PINK-1::GFP fluorescence reporter. GFP fluorescence was normalized to a constitutively-expressed RFP signal. Data presented in (A-F) are one representative result from four biological replicates. At least 150 worms were analyzed for each biological replicate (approximately 50 worms per image). Error bars in D, F represent SEM. Asterisks indicate *p*-value < 0.01, based on Student's *t*-test.

In experiments described above, we used pyoverdine-rich filtrate to ensure that these phenomena are representative of physiological pyoverdine concentrations. However, to confirm that the results observed are due to pyoverdine, we used established techniques ([29,30], see Methods for details) to purify pyoverdine. As expected, purified pyoverdine (but not iron-saturated purified pyoverdine) was capable of translocation into *C. elegans* pharynx (Fig 3A,B). Futhermore, purified pyoverdine exposure altered the NADH:NAD^+^ ratio (Fig 3C,D) and activated the mitophagy reporter PINK-1::GFP (Fig 3E,F).

### Pyoverdine triggers a distinct defense response in the host

**Figure 4.**
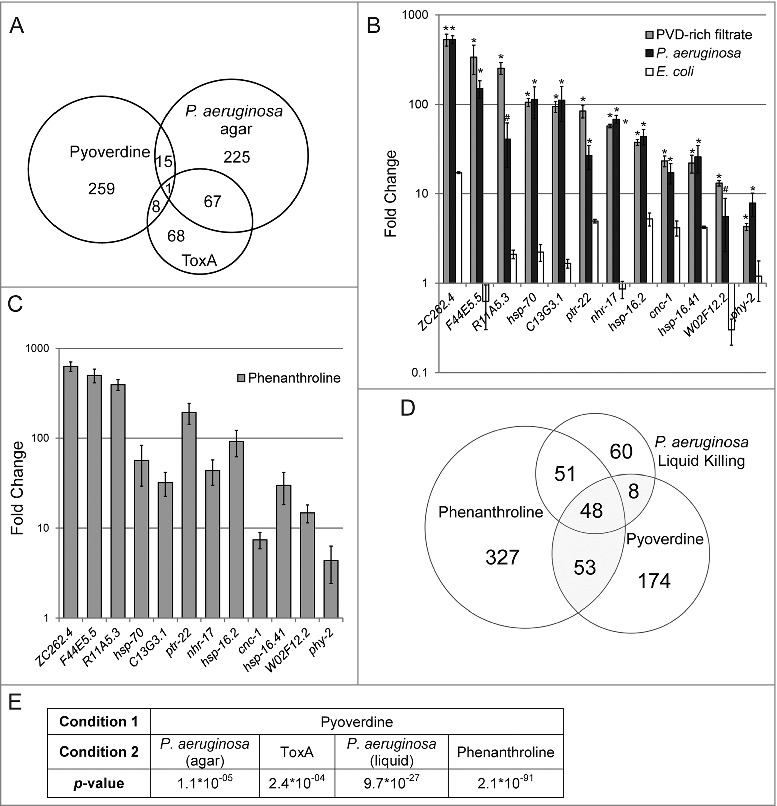
Pyoverdine exposure activates a distinct host response. (A) A Venn diagram of genes upregulated after exposure to pyoverdine and *P. aeruginosa* infection on agar and ToxA. (B) qRT-PCR analysis of a panel of genes upregulated by exposure to pyoverdine (gray), *P. aeruginosa* (black), or *E. coli* OP50 (white) in liquid as compared to untreated worms (which remained on NGM agar with *E. coli* OP50 food). (C) qRT-PCR analysis response to phenanthroline, with fold changes normalized to worms exposed to solvent control (DMSO) in liquid. (D) Venn diagram of genes upregulated after exposure to pyoverdine, *P. aeruginosa*, and phenanthroline in liquid. (E) Statistical significance of overlaps between microarray conditions in A and D based on hypergeometric probability. Asterisks in (B) indicate significant difference from *E. coli* OP50 (*p*-value < 0.01, based on Student's *t*-test). Error bars in B-C represent SEM of 3 independent biological replicates.

To obtain a more complete understanding of the consequences of pyoverdine exposure, we used microarray analysis to compare transcriptional profiles of worms exposed to 200 μM commercially-sourced purified pyoverdine in S Basal, S Basal alone, or untreated (left on NGM plates seeded with OP50). Afterward, upregulated genes from pyoverdine exposure were compared to genes upregulated after exposure to exotoxin A (a translational inhibitor produced by *P. aeruginosa*) or genes upregulated during infection with *P. aeruginosa* on agar plates (Table S1, Fig 4A) [31,32]*.*

Interestingly, while 68 genes were upregulated by both ToxA and *P. aeruginosa* infection (almost half of the genes upregulated by ToxA [31]), only 16 and 9 genes were in common between pyoverdine and *P. aeruginosa* infection on agar or ToxA, respectively. While statistically significant (See Fig 4E for *p*-values), these shared genes likely comprise a relatively non-specific generic stress response (Table S2).

This conclusion was bolstered by qRT-PCR analysis of 12 genes (selected on the basis of significant upregulation after pyoverdine exposure and to represent several classes of genes). Expression patterns for these 12 genes were assayed in worms treated with purified pyoverdine, pyoverdine-enriched filtrate, or *P. aeruginosa* under Liquid Killing conditions [4] (all normalized to OP50-fed *C. elegans* on agar media, Fig 4B). Expression of these 12 genes was also assayed in worms exposed to phenanthroline (Fig 4C), which were compared to worms exposed to DMSO (solvent).

In each case, the same expression pattern was observed, suggesting that the host is responding to perception of damage that is the same after each treatment. To perform an unbiased, genome-wide analysis, we compared transcriptome profiles of genes upregulated by pyoverdine (this study) to genes upregulated by exposure to either *P. aeruginosa* in liquid or to phenanthroline [33]. A statistically (and biologically) significant number of genes were differentially regulated under these conditions: of the 283 genes upregulated by pyoverdine, 109 were also activated by either *P. aeruginosa* in liquid or phenanthroline (Fig 4D).

To test whether the genes identified in the microarray have an effect on host survival during pyoverdine exposure, we used RNAi to knock down genes that were highly upregulated or downregulated in the presence of pyoverdine. Worm survival was then assayed after treatment with *P. aeruginosa* filtrate for 40 h. Disruption of multiple upregulated genes increased susceptibility to pyoverdine-mediated damage (Fig S7A, C, E). In general, RNAi of these genes had no discernible effect on the worms' survival in S basal, suggesting that they confer a protective response against pyoverdine. A small number of cases did exhibit non-specific increases in mortality in S Basal after RNAi, suggesting that these genes are necessary for proper survival in liquid contexts (Fig S7E). In contrast, knocking down most of genes downregulated after pyoverdine exposure had little impact on survival compared to RNAi vector control (Fig S7B, D, F). These findings suggest that genes upregulated in the presence of pyoverdine play valuable defense roles in the host.

We also observed statistically significant overrepresentation of genes containing an Ethanol and Stress Response Element (*p* = 1.85*10^−8^, see Table S3 for the list of genes). This motif has been linked to a master stress response pathway, conserved from *C. elegans* to mammals, that is activated by a wide variety of abiotic stresses [34,35,36,37,38]. More recently, the ESRE motif has been tied to mitochondrial surveillance [33,39], which is consistent with our assays showing that mitochondrial function is compromised after pyoverdine exposure. ESRE containing genes were also enriched in genes upregulated by *P. aeruginosa* in liquid [33].

Two larger implications arise from our observations regarding defense responses of *C. elegans* to the pathogen *P. aeruginosa*. First, the ability of the host to fine-tune its response to the mechanisms of virulence utilized by the pathogen, rather than triggering the same response regardless of context, shows remarkable complexity for an evolutionarily simple model organism. Second, the divergent responses strongly argue that the host does not simply trigger defenses based on structural determinants of the pathogen (pathogen-associated molecular patterns, or PAMPs). Instead, given the phenomena observed, it is more likely that the differences in gene expression are driven by damage-associated molecular patterns (DAMPs). To date, several different mitochondrial DAMPs are known, including mitochondrial DNA, *N*-formylated peptides, free cardiolipin, ATP, and several mitochondrial proteins [40,41,42]). Our data show that pyoverdine damages mitochondria and that a mitochondrial surveillance pathway, the ESRE pathway, is activated in response. Together, these data indicate that mitochondrial iron removal may be a heretofore unknown DAMP. Alternatively, the ESRE network may be activated by one or more of the previously known mitochondrial DAMPs, if they are released after pyoverdine intoxication. The answers to this question are currently under investigation.

### Conclusion

We have developed the following model to explain our observations. When available iron is limited, *P. aeruginosa* secretes pyoverdine to supplement its intracellular stores. This siderophore is ingested by *C. elegans*, along with other substances in its liquid milieu. Once within the host, pyoverdine, by an unknown mechanism, gains access to and removes ferric iron. A likely target for this abstraction is host mitochondria, which are iron-rich organelles. Removal causes significant damage, disrupting mitochondrial function and targeting mitochondria for turnover.

Due to its low solubility at near neutral pH, iron is generally not freely available to either host or pathogen, but is essential for cellular functions in each. Competition for iron is a crucial facet of host-pathogen interactions and has led to a molecular 'arms race' [43]. Pathogens developed sensitive systems to detect low intracellular iron and to promote siderophore production [44,45]. Susceptible hosts responded by developing iron-sequestering proteins in part to limit iron availability to pathogens [43]. As siderophores evolved to increase their iron affinity and gain the ability to extract iron from host proteins, hosts developed new defenses, such as siderophore-binding proteins (siderocalins) [46]. Although a few siderophores can liberate iron from host iron-sequestering proteins [43], where and how this occurs in vivo, and whether siderophores can enter cells remain important, and open, questions.

## Materials and methods

### Strains

*P. aeruginosa* strains:

*P. aeruginosa* PA14 [47]

*P. aeruginosa* PA14*pvdA* mutant [48]

*C. elegans* strains:

ALF25 (Peredox Reporter): P*myo-3*::Peredox::*unc-119* (IS) RED – this study

JJ1271 *glo-1(zu391)*

NVK90 (PINK-1::GFP reporter): *pink-1(tm1779); [Ppink-1::myc::pink-1::gfp; Pmyo-2::mCherry]* – this study

NVK204 (mtRosella): *glp-4(bn2);* P*myo-3::*mtRosella*::unc-119* (IS) RED [28]

PE327 (ATP reporter): *glp-4(bn2);* feIs5 [sur-5p::luciferase::GFP + rol-6(su1006)] [49]

SS104: *glp-4(bn2ts)* [50]

C. elegans – Pyoverdine/P. aeruginosa assays

Experiments with pyoverdine or *P. aeruginosa* and *C. elegans* were largely performed as previously described [51]. For experiments involving pyoverdine translocation, ∼6,000 worms/well in a final volume of 2 mL pyoverdine-rich filtrate in 6-well plates were used. For pyoverdine assays with live *P. aeruginosa*, worms were incubated with *P. aeruginosa* in Liquid Killing media [52].

Lysates were collected from ∼15,000 worms for each condition for each biological replicate. Worms were transferred into 15mL conicals and rinsed 4 times prior to freezing in identical volumes. Samples were thawed at room temperature and disrupted via sonication using a 2 min cycle on a Branson 250 Sonifier set to 70% output, 10% duty cycle. Pyoverdine in the worm lysate was measured spectrofluorometrically (Ex 405nm, Em 460nm) using a BioTek Cytation5.

To measure ferripyoverdine, 8-hydroxyquinoline was used to extract iron, which restores pyoverdine fluorescence [19]. In brief, solutions carrying ferripyoverdine were mixed in a 1:1 (v:v) ratio with 1M 8-hydroxyquinoline dissolved in chloroform. Samples were incubated at room temperature for 16h on a rotary mixer. Organic and aqueous (including pyoverdine) phases were separated by centrifugation.

### Worm lysate fractionation

About 15,000 worms were exposed to *P. aeruginosa* filtrate then extensively washed in S Basal buffer, frozen at -80°C, and subsequently thawed prior to sonication. After sonication, worm lysate was centrifuged at 2580 RCF for 20 min to pellet cell debris. Lysate supernatant was ultracentrifuged at 295,200 RCF for 4 h to pellet microsomes. Ultracentrifuged supernatant was collected as cytoplasmic content. Pyoverdine was measured fluorometrically (Ex405 Em 460) after 1 M 8-hydroxyquinoline treatment for 16 h.

### ICP-MS

For ICP-MS experiments, worms were prepared as for the measurement of internalized pyoverdine. In brief, approximately 45,000 *C. elegans* L1 larvae per sample were raised on NGM plates seeded with *E. coli* strain OP50. When they reached young adulthood, they were incubated in 6-well plates (2 mL/well) for 24h in pyoverdine-rich filtrate, *P. aeruginosa*, or for 16 h in 750µM ciclopirox olamine or 1,10-phenanthroline. After exposure, worms were transferred to 15 mL conicals, washed four times, and then collected with uniform volumes. Samples were frozen, thawed, and sonicated as described above. After lysis, samples were transferred to glass scintillation vials and water was evaporated by heating at 100°C on a heating block. Upon the loss of all visible moisture, samples were digested in 4 mL of concentrated, trace-metals grade nitric acid (GFS Chemicals) overnight at 98°C. For ICP-MS analysis, samples were resuspended in 1 mL concentrated nitric acid, diluted 1/40 into 2% HNO_3_ (v:v), 2% ethanol (v:v), and spiked with yttrium/indium to serve as internal standards. For iron quantitation, a seven point calibration curve from 3 ug/L to 200 ug/L was prepared in 2% HNO_3_ (v:v), 2% ethanol (v:v) and spiked with yttrium/indium internal standards. Linear correlation coefficients for the iron calibration curve were at least 0.9998.

Quantitation of iron was carried out by inductively coupled plasma mass spectrometry (ICP-MS) analysis, using a Perkin Elmer Nexion 300X ICP-MS system, operated in the kinetic energy discrimination (KED) mode. Iron was monitored at m/z 57. Yttrium and indium were used as internal standards and were monitored at m/z 89 and m/z 115, respectively. Quadrupole scanning parameters were set to 20 scans per read, 1 read per replicate, and 3 replicates per sample. The instrument was operated in the peak hopping mode with a dwell time of 50 ms. For KED mode, the cell gas pressure was set at 5, the RPa and RPq were set at 0 and 0.25 respectively. The detector was operated in dual mode. Plasma and auxiliary gas flows were set at 16 and 1.2 L/min respectively. Nebulizer gas flow was tuned at the time of analysis and typically ranged between 0.99 and 1.02 L/min. Plasma power was 1600 W. The peristaltic pump was operated at 20 rpm for analysis and 40 rpm for flushing. Read delay, flush, and wash times were 60, 90 and 120 seconds respectively.

### Measurement of host iron

Fluorometric determination of ferric iron was performed as follows. First, ∼15,000 worms were exposed to media, pyoverdine-rich filtrate, or analogous material from PA14*pvdA* for 24 h. Afterward, worms were extensively washed and lysates were made, as described above.

Next, a standard solution of pyoverdine, with fluorescence within linear range of detection by spectrometry (15,000 – 20,000 AU) was prepared. This material was mixed (1:1, v/v) with water, ferric iron, or the *C. elegans* lysates. Mixtures were incubated for 10 min, and then pyoverdine fluorescence was measured. The 1:1 stoichiometric relationship between iron and pyoverdine makes the difference between initial and final fluorescence equivalent to the iron remaining in the *C. elegans*. Total iron was also measured using ICP-MS, as described.

### Pyoverdine Purification

Pyoverdine was isolated from spent pyoverdine-rich bacterial media using minor modifications of previously published protocols [29,30]. In brief, *P. aeruginosa* strain PA14 was grown in M9 media (1% (w/v) Difco 5X M9 salts, 11.3 g/L iron-limited casamino acids, 0.4% glucose, 1mM CaCl_2_, 1mM MgSO_4_) for 20–22 hours at 37°C with agitation. Bacteria were removed by centrifugation and subsequent filtration through a 0.22 μm filter. Pyoverdine concentration in the resulting media was measured spectrofluorometrically; only samples with whose fluorescence oversaturated the detection limit of the machine were selected for further purification steps. Filtered, spent growth media was then autoclaved (121° C, 15 min) and ultracentrifuged (4h, 149,000 RCF). Supernatant was collected and frozen at -80° C prior to use.

For obtaining purified pyoverdine, the ultracentrifuged supernatant was passed over a C_18_ reverse-phase SepPak column, sequentially washed with 5 mL of H_2_O, 10 mL of 10% methanol, and 10mL 50% methanol, and eluted with 75% methanol. Methanol in the elution was evaporated. Presence of pyoverdine was measured spectrofluorometrically and confirmed by fluorescence quenching by concentrated ferric chloride solution.

### RNA purification, microarray and qRT-PCR

For microarray studies, worms were grown on NGM (nematode growth media) plates seeded with *E. coli* OP50 until young adulthood. About 6,000 worms were exposed to 200 µM commercially-provided purified *P. aeruginosa* pyoverdine in liquid S Basal media (EMC Microcollections, experimental condition), S Basal in liquid (first control condition), or left on NGM plates seeded with OP50 (second control condition) for 12h. Worms were then transferred into a 15 mL conical and washed twice with S Basal. Three biological replicates were tested for each condition. RNA was purified using Trizol extraction followed by RNA clean-up using an RNEasy kit (Qiagen). cRNA was prepared according to the manufacturer's protocol and hybridized to Affimetrix *C. elegans* GeneChip™ (Platform GPL200).

Gene expression was analyzed using GCRMA (http://www.bioconductor.org). Differentially upregulated genes were determined on the basis of fold change compared to both controls (>3) and the value of a modified Wilcoxon rank test >1.5 against both controls. The Wilcoxon coefficient was determined for each probeset as the smallest expression value amongst pyoverdine treatment replicates divided by the highest expression value in the control condition replicates. Microarray data were deposited in GEO database and are available using following link: https://www.ncbi.nlm.nih.gov/geo/query/acc.cgi?token = yrixumeohvwdpsb&acc = GSE95510.

For experiments involving quantification of expression of pyoverdine-dependent genes (qRT-PCR), the length of the treatment was 12h, compounds (phenanthroline or DMSO) were used at a final concentration of 50 µM. RNA collection was performed as for microarray analysis. At least 3 replicates were done per each condition. cDNA was synthesized using a RETROScript Kit (Ambion). *snb-1* was used as a housekeeping gene. Pyoverdine-dependent genes for qRT-PCR were chosen based on the following criteria: 1) substantial upregulation after pyoverdine exposure; 2) representation of different gene classes; 3) ability to design 20bp intron-spanning primer with 50% GC content. Fold-changes were calculated using a ΔΔCt method. *p*-values were calculated by Student's t test. Primer sequences are available upon request.

### Mitochondrial DNA quantification

Total DNA was extracted from a synchronized population of ∼6,000 worms washed from NGM plates seeded with *E. coli* OP50 and treated with filtrate produced by WT *P. aeruginosa* PA14 or PA14*pvdA* mutant. Primers were designed for the *nd1* mitochondrial gene and *act-3*, as previously described [3]. DNA was used for quantitative PCR using SYBR Green (BioRad), and mitochondrial gene level was normalized to nuclear gene number using the ΔΔCt method. Thermocycler parameters were as for qRT-PCR. At least three biological replicates were performed. *p*-values were calculated by Student's t test. Primer sequences are available upon request.

### Comparisons of microarrays

Pyoverdine-dependent genes were defined as described above. *P. aeruginosa*-dependent genes were taken from Troemel *et al.* [32] ToxA-upregulated genes were described in McEwan *et al.* [31]. Genes upregulated by *P. aeruginosa* in liquid and phenanthroline were taken from Tjahjono and Kirienko[33]. All these microarrays were performed using GPL200 platform (Affimetrix). All comparisons were performed between upregulated genes only. Area-proportional Venn diagrams were drawn using the eulerAPE (http://www.eulerdiagrams.org/eulerAPE/v2/).

Statistical significance of overlaps between microarray conditions was determined based on hypergeometric probability.

### Construction of Peredox reporter transgenic worms

The coding sequence for the Peredox NADH-sensitive ratiometric reporter was PCR-amplified from the pcDNA3.1-Peredox-mCherry plasmid (Addgene #32383) and subcloned into the pPD93.97 plasmid (Addgene #1476) which uses the *myo-3* promoter to drive Peredox expression in the body wall muscles [25]. To construct an mCherry-expressing transgene for normalization of Peredox expression, the mCherry sequence from pPD95.79 mCherry was subcloned into pPD93.97 as a KpnI-ApaI restriction fragment [53]. The mCherry expressing transgene was then retrofitted with the *unc-119* coding sequence from *Caenorhabditis briggsae* as previously described [54]. The Peredox and mCherry-expressing plasmids were then purified using a Qiagen kit, and mixed for bombardment of the DP38(*unc-119(ed3)*) strain following an established protocol [55]. From the bombarded animals, an intergrated line showing expression of both Peredox and mCherry was identified and then outcrossed 4-times with wild-type N2 worms to produce ALF25 (*baf25(myo-3::Peredox, myo-3::mCherry, unc-119(+))*).

### Generation of muscle-expressed mtRosella strain

The plasmid expressing mtRosella under the control of the *myo-3* promoter was graciously shared by the Tavernarakis lab [56]. Since existing strains carrying the transgene used *rol-6* as an injection marker, the plasmid was retrofitted to carry the *unc-119* gene from *Caenorhabditis briggsae* using a cassette from the *punc-119cbr* plasmid as previously described [54]. The purified plasmid was then used to bombard the HT1593 (*unc-119(ed3)*) via biolistic bombardment [55], and a line with an integrated transgene producing visible fluorescence was identified. After outcrossing, this strain was designated ALF89 (*bafIs89*). This strain was crossed in g*lp-4(bn2)*, resulting in NVK204.

### Mitochondrial Health Reporter Assays

For PINK1-GFP and cytosolic NADH/NAD^+^ ratiometric reporter assays, approximately 6,000/well young adult worms were incubated for 24h in 6-well plates containing one of the following: filtrate from *P. aeruginosa* PA14, filtrate from *P. aeruginosa* PA14*pvdA*, wild-type filtrate preincubated with iron, purified pyoverdine from wild-type *P. aeruginosa* PA14, or media alone (6h exposure for purified pyoverdine). Worms were then transferred to 96-well black, clear-bottom plates (Greiner) and washed in S Basal five times. For visualization of PINK-1::GFP or Peredox-GFP, young adult worms carrying the reporters were exposed to media or purified pyoverdine in 96-well plates. A Cytation5 multimode reader (BioTek Instruments) GFP (Ex 469, Em 525) and RFP (Ex 531, Em 593) fluorescence filters were used to collect each image. For each strain, imaging was performed with identical settings for the control and treatment.

For determination of ATP concentrations, approximately 6,000 young adult worms/well were incubated for 24h in 6-well plates containing either filtrate from *P. aeruginosa* PA14 or media alone. The worms were transferred to 96-well, white clear bottom plates and washed in S Basal five times. 150µL of Luminescence buffer (0.14M K_2_PO_4_, 0.03M sodium citrate, 1% DMSO, 1mM luciferin) was added to each well in the plate [49]. Bioluminescence and GFP fluorescence was measured every 5 minutes for 6h, and 1 h after addition of luciferin was chosen as a representative time point. Due to the first-order kinetics of luciferase, luminescence in the presence of excess luciferin is directly proportional to ATP concentration.

### Confocal microscopy

To detect pyoverdine inside *C. elegans, glo-1(zu391)* or *glp-4(bn2)* worms were exposed to pyoverdine-rich *P. aeruginosa* filtrate, pyoverdine-deficient filtrate from *P. aeruginosa pvdA*, uninoculated media, or pyoverdine-rich filtrate supplemented with FeCl_3_ or Ga(NO_3_)_3_ for 16h. FeCl_3_ was added until no fluorescence was detected in the filtrate. The concentration of Ga(III) added was identical to that of Fe(III). After exposure, worms were extensively washed in S Basal buffer, paralyzed with 25mM levamisole hydrochloride, and mounted on slides with 3% noble agar. Images were acquired with a Ziess LSM710 confocal laser-scanning microscope using a 405nm argon laser for excitation of pyoverdine fluorescence. All images were acquired under identical conditions.

For the mtRosella mitophagy reporter assay, approximately 6,000 healthy young adult worms were added to each well of a 6-well plate. Worms were incubated for 24h with filtrate from *P. aeruginosa* PA14, filtrate from *P. aeruginosa* PA14*pvdA*, wild-type filtrate preincubated with iron, or media alone. After exposure, worms were extensively washed in S Basal buffer, paralyzed with 25mM levamisole hydrochloride immediately prior to imaging, and mounted on 3% noble agar. Images were acquired with a Ziess LSM710 confocal laser-scanning microscope using a 488nm laser for excitation of GFP and 543nm laser for excitation of DsRed. All images were acquired under identical conditions.

## Supplementary Material

Kang_etal.zip
