# Supplementary material for: Pyoverdine, a siderophore from Pseudomonas aeruginosa, translocates into C. elegans, removes iron, and activates a distinct host response
Source: Virulence. 2018 Apr 24;9(1):804–17. doi: 10.1080/21505594.2018.1449508 (PMC5955448; doi:10.1080/21505594.2018.1449508)
Supplement: Kang_etal.zip [file kvir-09-01-1449508-s001.zip › Kang etal/Kang et al Supplementary Material.pdf]

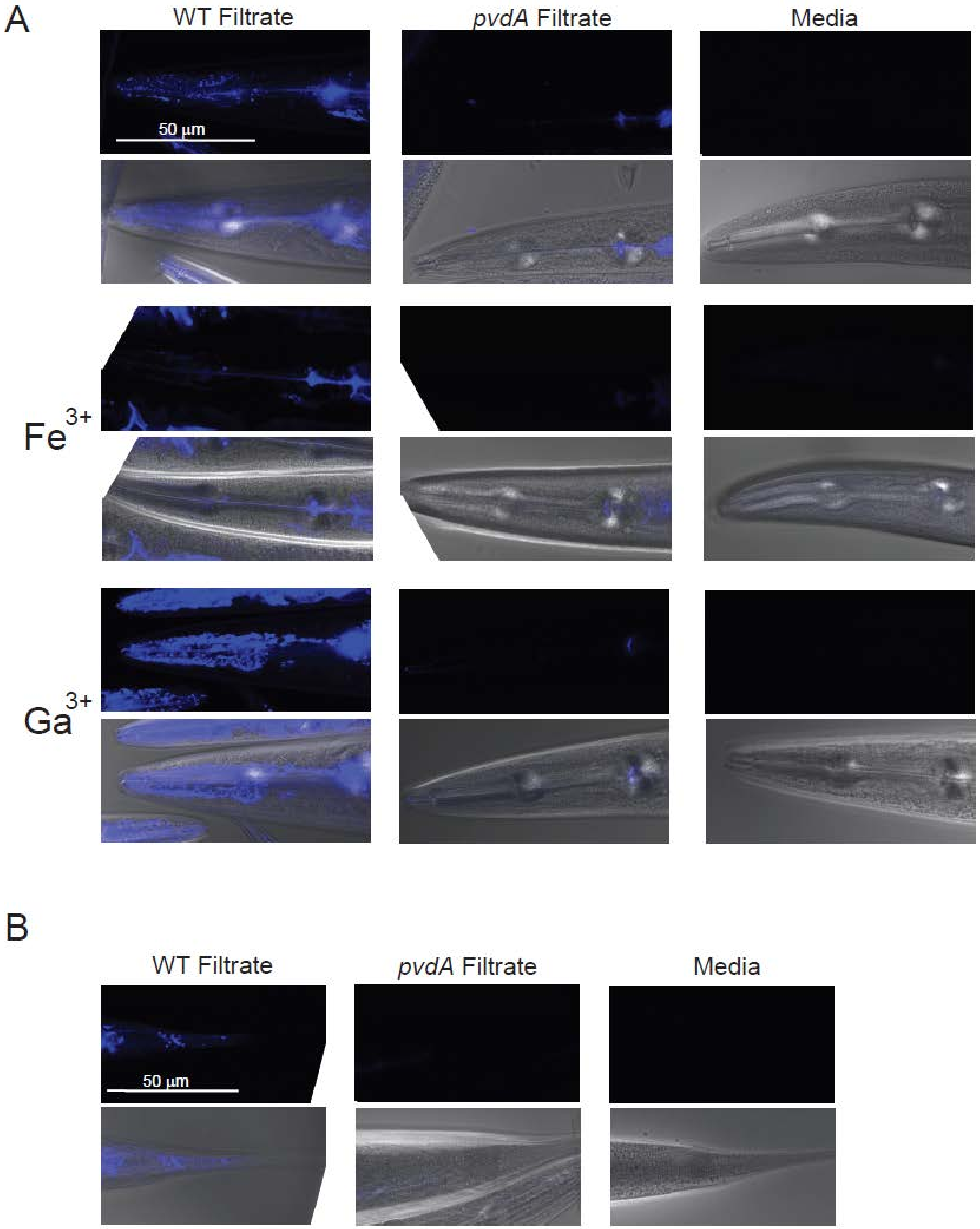

Figure S1. Pyoverdine enters *C. elegans*.

(A) Confocal laser scanning micrographs of the pharyngeal region of *C. elegans* exposed to pyoverdine-rich bacteria free filtrate, identical material from a pyoverdine-deficient mutant *pvdA*, or media alone (top). Images of the pharyngeal region of *C. elegans* were also collected after exposure to the same filtrates were pre-incubated with ferric iron (middle) or gallium (bottom). (B) Confocal laser scanning micrographs of the tail region of *C. elegans* after exposure to pyoverdine-rich bacteria free filtrate, identical material from a pyoverdine-deficient mutant *pvdA*, or media alone. All images were collected using identical settings.

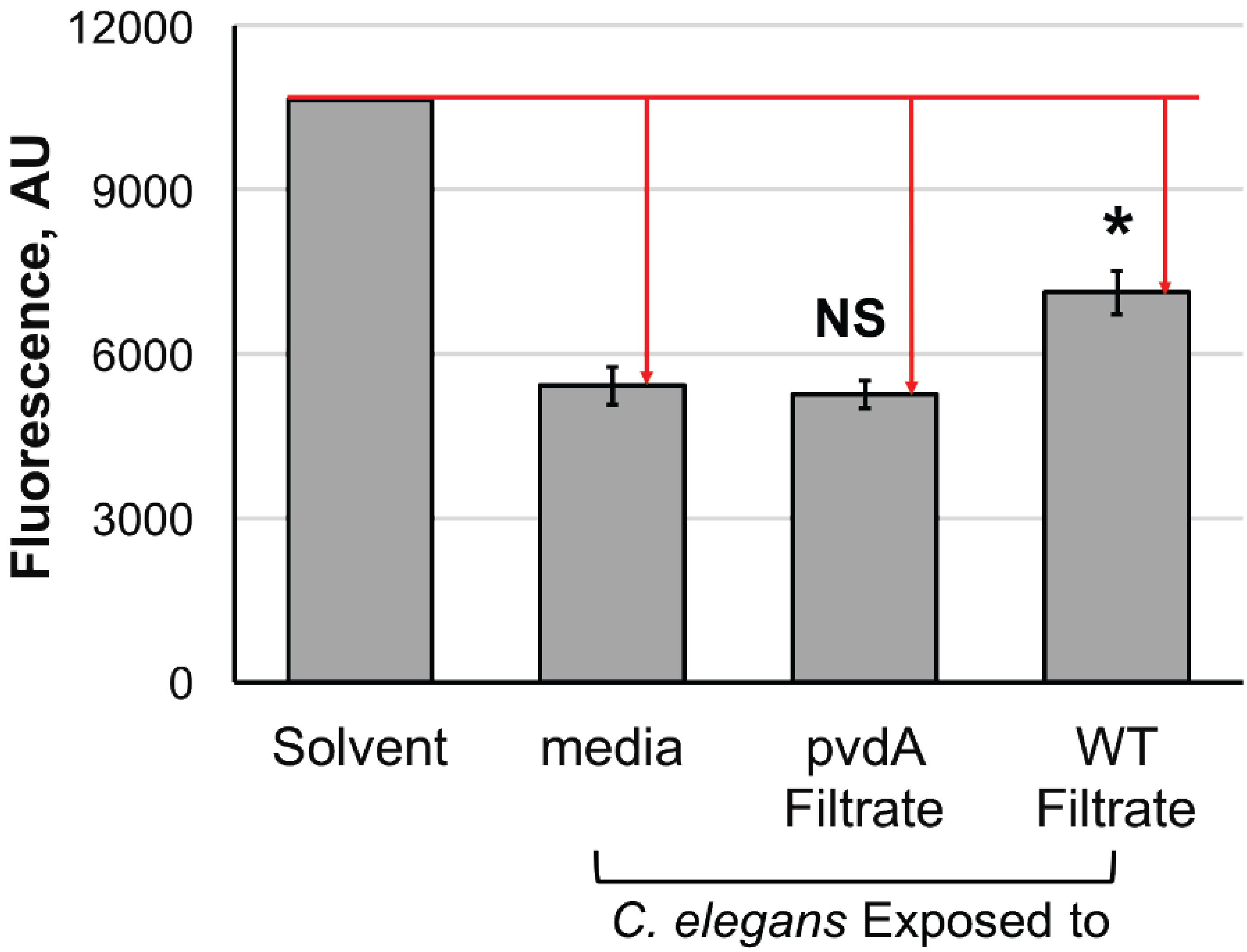

Figure S2. Pyoverdine exposure decreases host ferric iron content.
Spectometric measurements of pyoverdine fluorescence after addition of S Basal (left) or lysates from worms exposed to media (center left), pyoverdine-deficient filtrate from a PA14*pvdA* mutant (center right), or pyoverdine-rich filtrate from wild-type PA14 (right). The difference in resultant fluorescence in the latter three samples represents quenching due to iron present in the media or lysates. Error bars represent SEM of 3 biological replicates. Asterisk represents significant difference ($p < 0.05$) compared to media control using Student's *t*-test, NS corresponds to $p > 0.05$ compared to media.

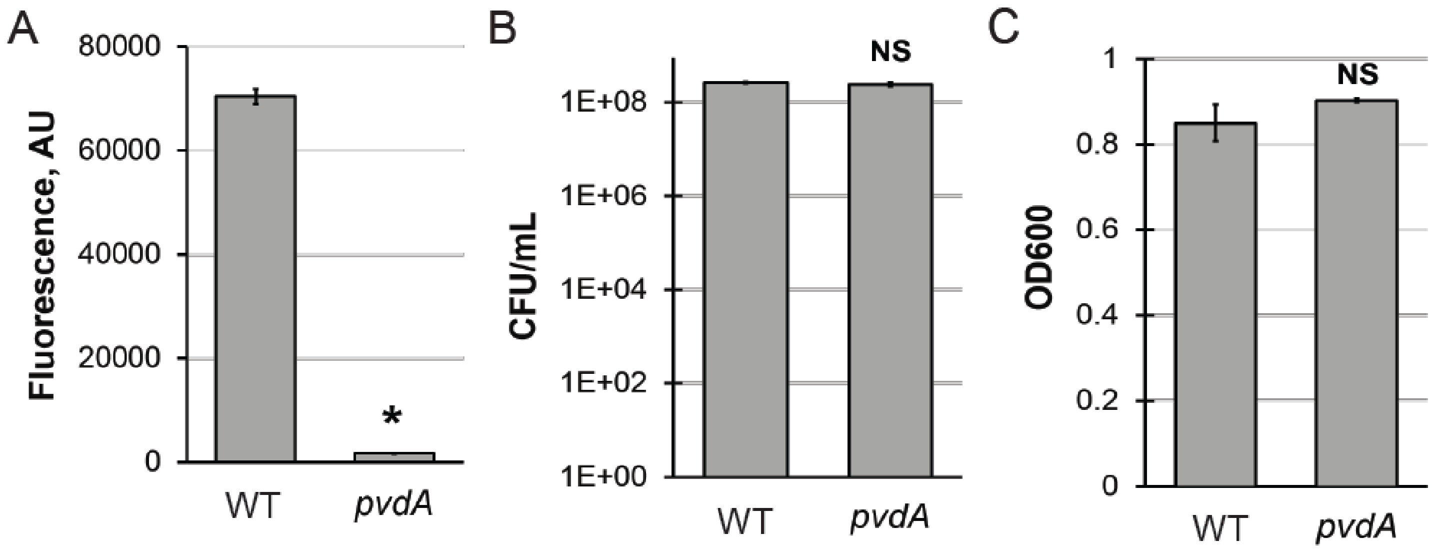

Figure S3. Growth of PA14*pvdA* is normal in M9 media.
(A) Pyoverdine production by wild-type *P. aeruginosa* PA14 and PA14*pvdA* after 24 h aerobic-shaking incubation in M9 media. (B, C) Bacterial density of the two cultures measured in colony forming units per mL (B) or optical density at 600 nm (C). For all data presented, error bars represent SEM of 3 biological replicates. Asterisks indicate significant difference between conditions ($p < 0.01$) based on Student's *t*-test. NS corresponds to $p>0.05$, based on Student's *t*-test.

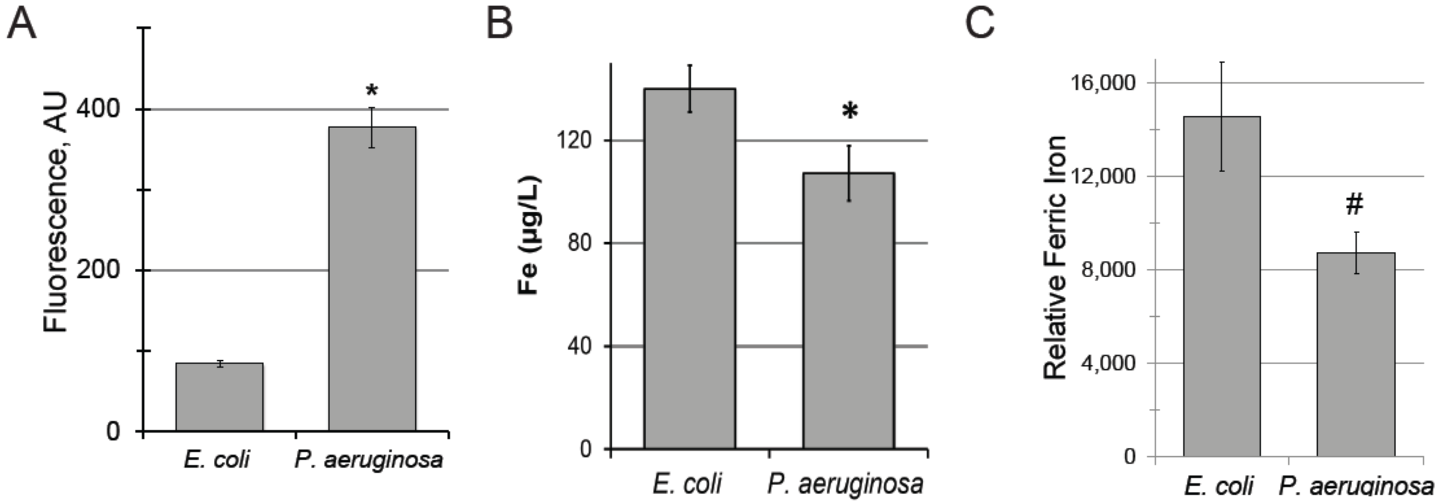

Figure S4. Exposure to *P. aeruginosa* in liquid results in pyoverdine translocation and host iron removal.
(A) Quantification of pyoverdine in *C. elegans* exposed to *P. aeruginosa* PA14 in liquid media for 36 h. **(B)** ICP-MS analysis of iron concentration in worms exposed to *P. aeruginosa* PA14 in liquid media. **(C)** Fluorometric measurement of ferric iron in *C. elegans* exposed to either *E. coli* OP50 or *P. aeruginosa* PA14. For all data presented, error bars represent SEM of 3 biological replicates. Asterisks indicate significant difference between conditions ($p < 0.01$) based on Student's *t*-test.

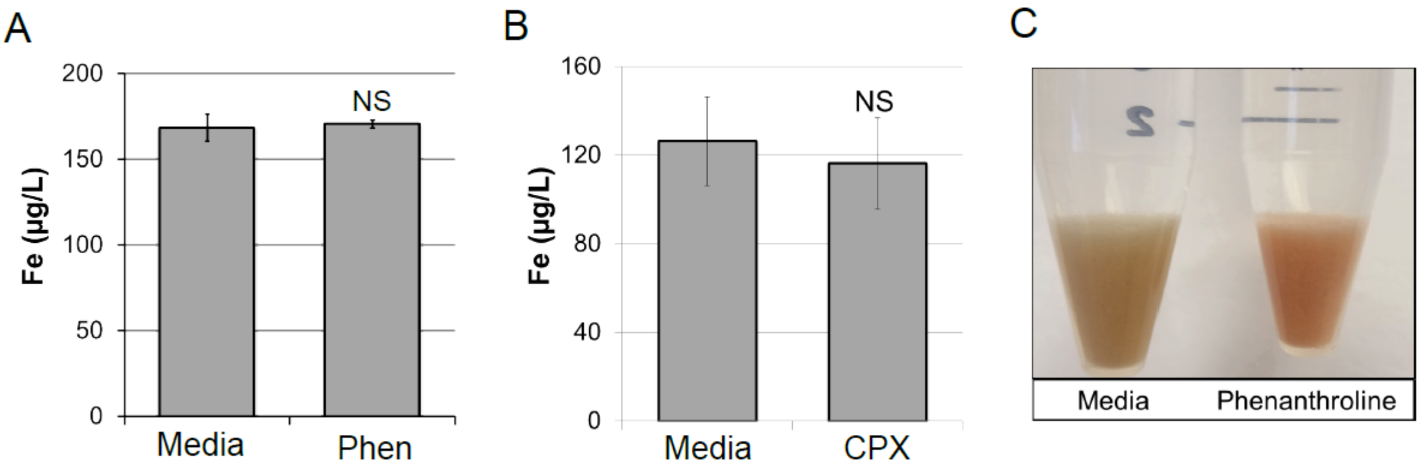

Figure S5. Small molecule iron chelators do not reduce host iron content.
(A) ICP-MS analysis of iron concentration in worms exposed to media control or to 750µM 1,10-phenanthroline (Phen). **(B)** ICP-MS analysis of iron concentration in worms exposed to media control or to 750µM ciclopirox olamine (CPX). **(C)** A photograph of *C. elegans* after exposure to media control or to 750µM 1,10-phenanthroline shows the reddish hue of the iron:1,10-phenanthroline complex. Error bars in **A-B** represent SEM between averages of three biological replicates. NS corresponds to $p>0.05$, based on Student's *t*-test.

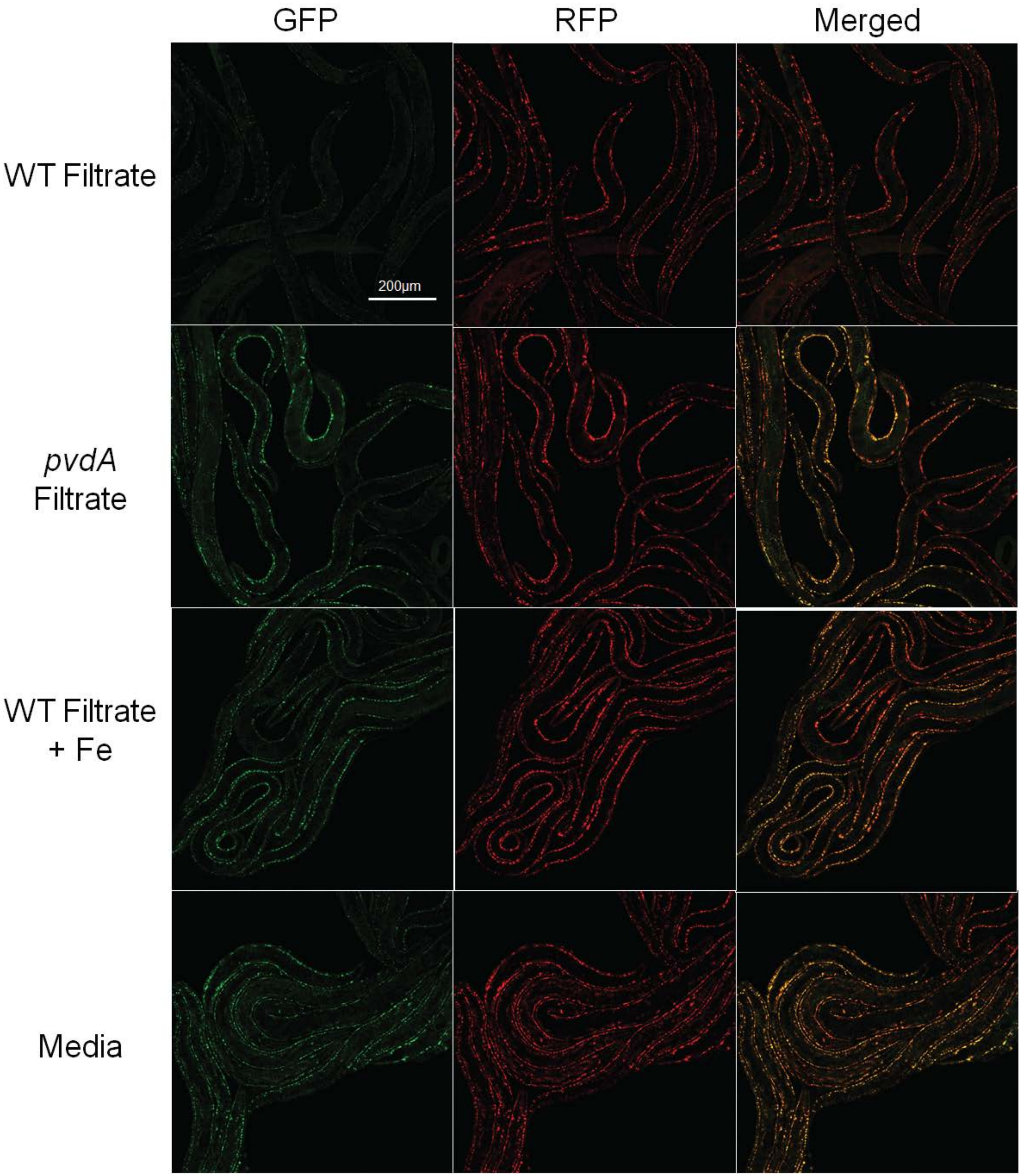

Figure S6. Exposure to pyoverdine triggers mitochondrial degradation.

Low-magnification micrographs of worms containing the mtRosella reporter. Worms were treated with WT filtrate, identically-prepared from a pyoverdine biosynthesis mutant (PA14*pvdA*), wild-type filtrate pre-saturated with iron, or media alone. Fluorescence of GFP requires proper protein folding, which is unstable at low pH values (such as those in autophagolysosomes). DsRed folding is stable at this pH.

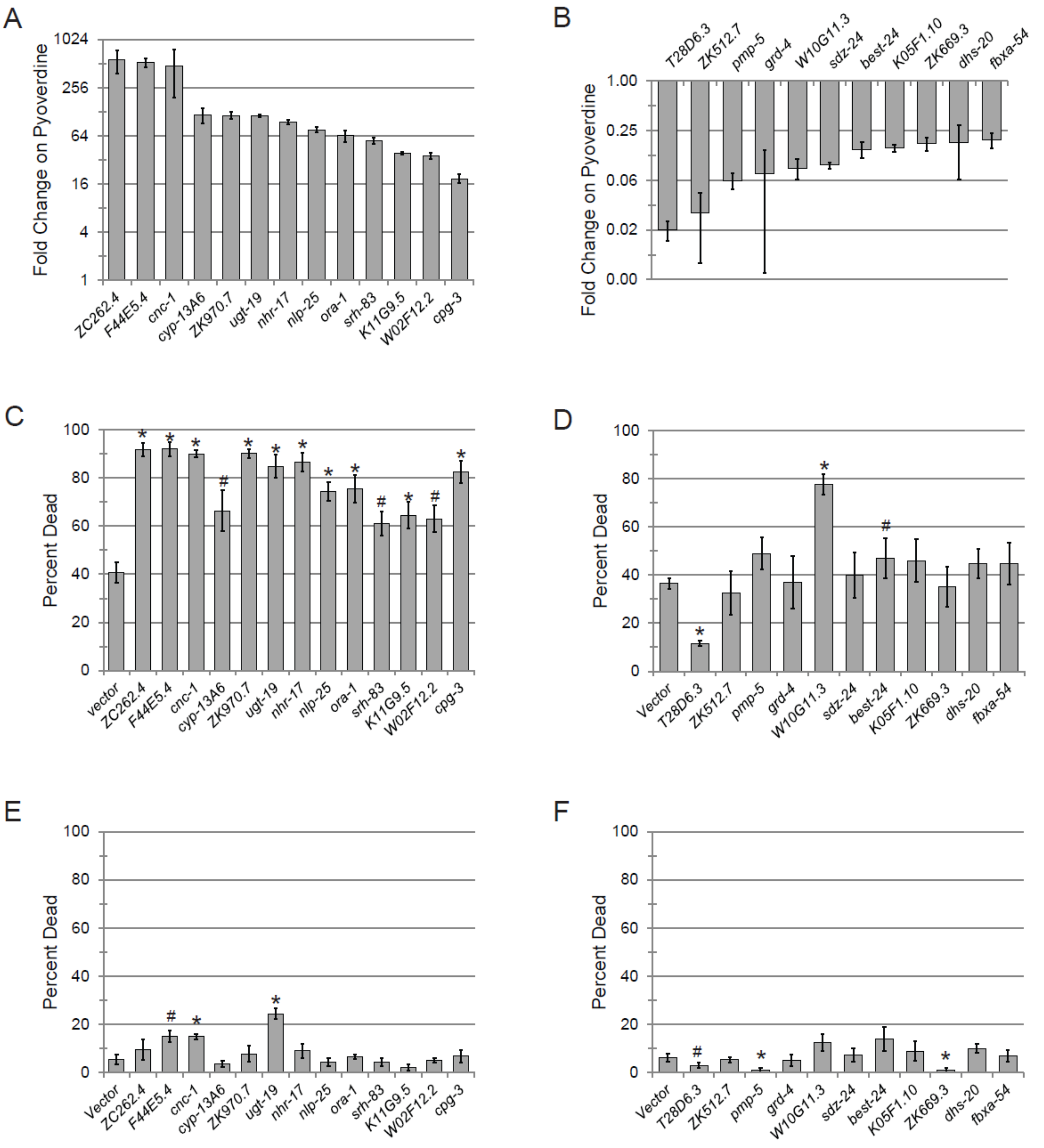

Figure S7. Genes upregulated in the presence of pyoverdine are important for host defense.
(A, B) qRT-PCR analysis of genes up- **(A)** or down-regulated **(B)** in the presence of pyoverdine. **(C, D)** Death of *C. elegans* treated with RNAi toward genes up- **(C)** or down- regulated **(D)** fraction death after 40 h exposure to pyoverdine-rich filtrate from *P. aeruginosa* PA14. **(E, F)** Death of *C. elegans* treated with RNAi toward genes up- **(E)** or down- regulated **(F)** fraction death after 40 h S basal.

Movie S1. 3D reconstructed videos of worm pharynx after pyoverdine exposure.

glp-4(bn2) mutant worms were exposed to wild-type filtrate, filtrate from PA14*pvdA*, or wild-type filtrate pre-saturated with iron, then subjected to confocal laser scanning microscopy. z-stack images were reconstructed into three dimensional representations using ZEN image analysis software. Red fluorescence represents CellMask plasma membrane stain (Invitrogen, California) used to label pharyngeal cells.

Table S1. List of genes upregulated by pyoverdine exposure.

Genes upregulated by 12h exposure to pyoverdine in liquid (as compared to solvent in liquid and untreated worms on agar plates). Upregulation was significant compared to both controls; fold changes shown are normalized to the solvent in liquid condition.

Table S2. Genes upregulated by pyoverdine intoxication and infection with *P. aeruginosa* on agar or intoxication with ToxA.

Affymetrix probeset identifiers, wormbase IDs, gene identifiers, difference in expression, and functional attribution for genes upregulated both by exposure to pyoverdine and by infection with *P. aeruginosa* infection on agar (top) or ToxA exposure (bottom). Genes upregulated in all three conditions are shown in bold.

Table S3. ESRE-containing genes upregulated by pyoverdine intoxication.

Affymetrix probeset identifiers, wormbase IDs, and gene identifiers for genes upregulated by pyoverdine intoxication that contain an ESRE motif within their promoter region.

Table S2. List of genes, upregulated by pyoverdine and *P. aeruginosa* or ToxA.

Probe Set ID	WB ID	Gene	Name	Fold	Description
**Genes, upregulated by pyoverdine and *P. aeruginosa* on agar**					
190585_at	WBGene00014173	ZK970.7	ZK970.7	118.1	hypothetical protein
172790_x_at	WBGene00008519	F02C12.5	*cyp-13B1*	51.5	Putative cytochrome P450 cyp-13B1
172437_x_at	WBGene00000556	R09B5.3	*cnc-2*	30.5	CaeNaCin (Caenorhabditis bacteriocin
**177903_s_at**	**WBGene00008602**	**F09B9.1**	***oac-14***	**27.0**	**O-ACyltransferase homolog**
184714_s_at	WBGene00021818	Y53G8B.2	Y53G8B.2	14.0	hypothetical protein
189578_at	WBGene00011672	T10B9.2	*cyp-13A5*	13.6	Putative cytochrome P450 CYP13A5
184624_s_at	WBGene00016119	C25H3.10	C25H3.10	12.4	hypothetical protein
187969_s_at	WBGene00003561	F02E8.6	*ncr-1*	7.4	Niemann-Pick C1 protein homolog 1
184191_at	WBGene00000558	R09B5.9	*cnc-4*	7.0	CaeNaCin (Caenorhabditis bacteriocin)
191543_at	WBGene00015455	C04G6.5	C04G6.5	5.3	UPF0057 membrane protein
183381_at	WBGene00016845	C50F7.5	C50F7.5	4.8	hypothetical protein
183815_at	WBGene00017667	F21E9.3	*ttr-37*	4.2	TransThyretin-Related family domain
189264_s_at	WBGene00016147	C26F1.2	*cyp-32A1*	3.7	CYtochrome P450 family
175519_at	WBGene00010328	F59C6.11	F59C6.11	3.7	hypothetical protein
189971_at	WBGene00001158	F01G10.3	*ech-9*	3.3	Enoyl-CoA Hydratase
184546_s_at	WBGene00016417	C34F11.8	C34F11.8	3.2	hypothetical protein
**Genes, upregulated by pyoverdine and ToxA**					
**177903_s_at**	**WBGene00008602**	**F09B9.1**	***oac-14***	**27.0**	**O-ACyltransferase homolog**
192924_s_at	WBGene00003844	K11C4.4	*odc-1*	17.5	Ornithine decarboxylase
193679_s_at	WBGene00003602	H01A20.1	*nhr-3*	16.9	Nuclear hormone receptor family member
174023_at	WBGene00003610	ZC410.1	*nhr-11*	12.1	Nuclear Hormone Receptor family
183310_s_at	WBGene00020725	T23C6.3	*cnp-3*	7.8	CalciNeurin binding Protein
185023_at	WBGene00013244	Y56A3A.33	Y56A3A.33	3.8	hypothetical protein
184744_at	WBGene00017901	F28E10.2	*igeg-1*	3.8	IG (immunoglobulin), EGF and transmmembrane domain
190554_at	WBGene00010700	K09A9.1	*nipi-3*	3.5	A kinase similar to the human kinase Tribbles homolog 1
180855_s_at	WBGene00003907	C52B9.7	*paf-2*	3.2	Platelet-activating factor acetylhydrolase homolog 2

Gene in bold (*oac-14*) was upregulated under all 3 conditions. Descriptions were provided by DAVID ID Conversion Tool.
